# A prospective cohort study: platelet-rich plasma combined with carpal tunnel release treating carpal tunnel syndrome

**DOI:** 10.1186/s12891-022-05733-8

**Published:** 2022-08-17

**Authors:** Yan-chun Gao, Qi-yang Wang, Chen-chen Wang, Shichang Zhao, Hua Chen

**Affiliations:** 1 Department of Orthopedic Surgery, Shanghai Jiao Tong University Affiliated Sixth People’s Hospital, Shanghai Jiao Tong University, Shanghai, 200233 China; 2 Shanghai Institute of Technology, Shanghai, 201418 China

**Keywords:** BTCQ –FSS, BCTQ-SSS, Carpal tunnel release (CTR), Carpal tunnel syndrome(CTS), Platelet-Rich Plasma (PRP), VAS

## Abstract

**Background:**

PRP injection was proved to promote the health condition of individuals with mild to moderate Carpal Tunnel Syndrome (CTS). However, carpal tunnel release (CTR) was still a necessary treatment for individuals with moderate and severe CTS.

**Methods:**

To explore whether adjuvant PRP treatment would improve the prognosis while using CTR, we included 82 patients in this study. Preoperative and postoperative visual analog scale (VAS), Boston carpal tunnel syndrome questionnaire-symptom severity scale (BCTQ-SSS), Boston carpal tunnel syndrome questionnaire-functional status scale (BCTQ-FSS), and grip strength were used to examine the patient's symptoms and function.

**Results:**

CTR combined with PRP treatment improved the VAS (1.9 ± 0.5 versus 1.4 ± 0.4, *P* < .05), BCTQ-SSS (1.8 ± 0.4versus 1.5 ± 0.3, *P* < .05) and BCTQ-FSS (1.8 ± 0.5 versus 1.4 ± 0.6, *P* < .05) in patients with moderate symptoms within one month after surgery. At the same time, it does not show any advantages in treating individuals with severe carpal tunnel syndrome.

**Conclusions:**

PRP does not affect long-term prognosis while increasing the surgery cost. To conclude, PRP as an adjuvant treatment of CTR has limited effect. Considering the additional financial burden on patients, CTR combined with PRP should be cautious in CTS treatment.

## Background

Carpal tunnel syndrome (CTS) constitutes the most joint compressive neuropathy of the upper limbs, usually via the abnormal flexor retinaculum thickening [1]. Pain, numbness, and muscle weakness caused by CTS seriously affect patients’ function and quality of life [2].

The median nerve is not isolated but entirely connected to myofascial structures. The perineurial area is in continuity with the deep fasciae of the forearm, suggesting that an unbalanced tension of epimysial fasciae can affect the perineural area, limiting nerve displacement. Consequently, this must be included in CTS pathogenesis [3, 4]. Although splinting and corticosteroid injections were proven adequate, strong evidence supported that the carpal tunnel release (CTR) decompresses the median nerve by dividing the transverse carpal ligament and should have a better treatment advantage at 6 and 12 months, especially in patients with moderate or severe symptoms [2, 5, 6].

Platelet-rich plasma (PRP) has been utilized as a safe treatment form in divergent settings [7]. It is an analogous biologic agent constituting concentrated platelets, the primary component of which is believed to be products of degradation consisting of transforming growth factor (TGF), the insulin-like growth factor-1 (IGF-1), Platelet-derived growth factor (PDGF), vascular endothelial growth factor (VEGF), and epidermal growth factor (EGF) [8]. Most research supported that PRP injection enhanced the clinical outcomes of individuals with mild-to-moderate CTS [9]. Research based on in vitro and in vivo studies supported the neurotrophic impact of PRP in peripheral nerves [10, 11]. Diverse growth factors are released and activated following a PRP injection, leading to median nerve rejuvenation and improving neural blood via protection of the blood-nerve barrier [12]. There is limited clinical information on the utilization of PRP in peripheral neuropathies in humans [9, 13]. A recent systematic review indicated that PRP is effective for individuals with mild to moderate carpal tunnel syndrome. At the same time, PRP injection alone is not recommended for severe carpal tunnel syndrome patients [9]. No studies have pointed out whether adjuvant PRP infusion is necessary for patients with severe symptoms who require surgical treatment.

For the lack of correlational studies, we tried to explore whether adjuvant PRP treatment could improve the prognosis of individuals with moderate to severe carpal tunnel syndrome while applying CTR. Further exploration is needed to provide strong research evidence by which surgeons should establish guidelines for utilizing this treatment in CTS patients.

## Patients and methods

### Ethical approval

This study is a prognostic study constituting a prospective cohort. The ethics committee at Shanghai Sixth People’s Hospital approved the study. In addition, this study was conducted per the Code of Ethics of the World Medical Association (Declaration of Helsinki) for human procedures.

### Patients

Between 2015 and 2018, 94 patients underwent CTR for CTS treatment in our hospital. Twelve patients declined to participate, and we included the remaining 82 patients in this study. Patients were divided into control and PRP groups according to whether adjuvant PRP was used during the procedure. There were 39 in the control group and 43 in the PRP group. All subjects included in the study had moderate to severe symptoms or failed conservative treatment with steroid injections [14]. Patients were excluded if they aged > 70 or < 18, with a course of CTS is less than two months, had space-occupying lesions within the carpal tunnel, had a pregnancy, diabetes mellitus, rheumatoid arthritis, traumatic CTS, or had previous CTS surgery. Before the operation, every patient underwent a nerve conduction study for definite diagnosis and severity evaluation [15]. According to a guideline published by Mooar, P. A. et al., the assessment of the severity of carpal tunnel syndrome is performed by an experienced hand surgeon (H Chen) [16]. 1. Low Severity (nighttime pain/sensory disturbances and/or episodic/infrequent symptoms) 2. Moderate Severity (pain/sensory disturbances, tingling, frequent activity-related symptoms, and/or difficulty with fine motor coordination) 3. High Severity (constant sensory loss, motor clinical findings, and/or thenar atrophy).

The demographic data consist of age, body mass index (BMI), and gender, along with preoperative evaluation by visual analog scale (VAS), Boston carpal tunnel syndrome questionnaire-symptom severity scale (BCTQ-SSS), Boston carpal tunnel syndrome questionnaire-functional status scale (BCTQ-FSS) and grip strength. Follow-up was performed at one, three, and six months after surgery. The CTS diagnosis was centered on the typical history constituting pain, sensory disturbances, and weakness entailing the median nerve distribution. The study included 35 males and 47 females. The mean age was 39.4 ± 10.1 years and BMI 24.9 ± 4.9. The mean symptom duration was 17.0 months, and the affected side was 46 on the right and 36 on the left.

### Operative procedure

Experienced surgeons performed all surgical procedures (YC Gao, QY Wang, H Chen). Regarding the conventional method, the approach documented by Taleisnik was applied [17]. In brief, a palmar longitudinal incision, starting at the ring finger axis, passed through the thenar and hypothenar eminences and proceeded proximally into the wrist proximal flexor crease. Following the exposure of the underlying transverse carpal ligament, its ulnar region was longitudinally cut. The median nerve was identified for protection, and the incision was closed routinely.

**Fig. 1:**
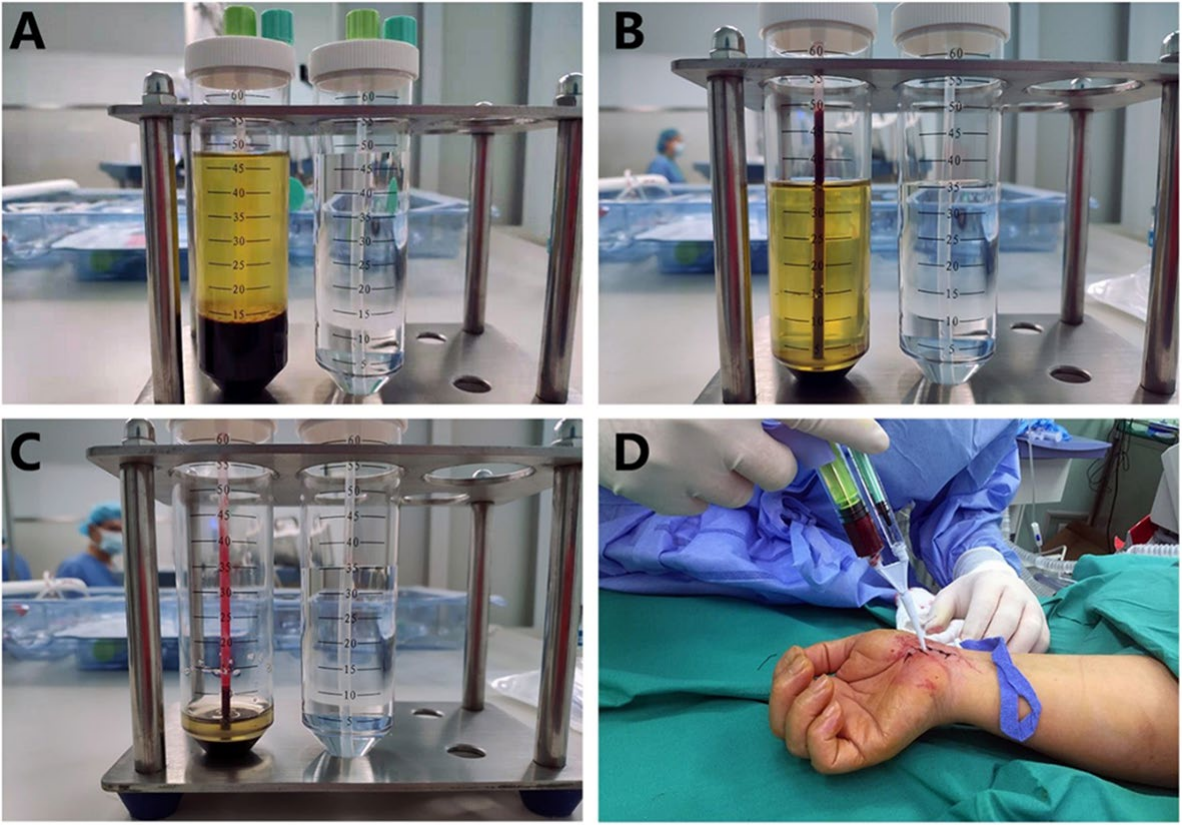
Preparation and injection of PRP. **A** Blood after the first centrifugation **B** Erythrocyte-depleted plasma **C** Platelet-rich plasma **D** Injection of platelet-rich plasma

At the time of surgery, 15 mL of analogous venous blood sample was drawn from every subject's contralateral (non-impacted) hand, followed by centrifugation at two successive density gradient centrifugations (800 g-10 min and 1100 g-10 min) at our hospital laboratory. In the initial centrifugation, separation of the red blood cells was achieved. In contrast, in the second centrifugation, separation of PRP from the platelet-poor plasma (PPP) and then introduced into a sterile injector. 2 mL PRP was injected from the sutured incision. Then the incision was bandaged (Fig. 1).

No brace or splint was used following the operation. Restricted wrist movement was permitted for 24 h. We encouraged the patients to return to their daily activities. The use of NSAIDs was restricted in both groups, and patients were advised that they did not require long-term use of NSAIDs to treat carpal tunnel syndrome.

### Outcome measures

Postoperative follow-up visits were held by a third physician (blinded to the groups) after 3 and 6 months, during which the VAS, BCTQ, and grip strength were repeated. Digital pain severity and paresthesia were determined via VAS, with 10 points designated as highly severe pain, while 0 points established no pain. BCTQ constitutes a symptom severity scale (SSS) and a functional status scale (FSS). The lower scores on the BCTQ imply lesser symptom severity and better active status of the patient [18]. The grip’s strength was determined with the flexion of the elbow at 90 degrees and in neutral rotation for the forearm.

Six months after the operation, the remission was evaluated according to the patient’s clinical symptoms in the last two weeks. Remission was defined as the significant reduction of symptoms, including numbness, pain, sensory disturbance, and muscle weakness. Patients with no preoperative symptoms were defined as entirely asymptomatic.

### Statistical analyses

Continuous variables were indicated as mean ± standard deviation; categorical data were shown as a number (percentage). For a constant variable, demographic data were assessed via the independent t-test for continuous data, while the X^2^ test was employed for the categorical data. The repeated-measures ANOVA followed by post hoc tests was carried out for the data at various follow-ups. All statistical analysis was conducted using SPSS 22.0 in this study(by CC Wang). All statistical evaluations were two-sided. *P* < 0 0.05 signified statistical significance.

## Result

Eighty-two patients (35 males and 47 females) were enrolled for analysis. All subjects were followed up for more than six months. In our cases, 35 of the 82 patients were male, and the average age of this series was 42.1 ± 11.7 (19 to 69). The right hand was affected in 46, and the left was involved in 35 cases. The mean BMI was 25.6 ± 4.1 in control group and 25.0 ± 5.1 in PRP group. The mean symptom duration was24.3 ± 21.7 months in control group and 26.8 ± 23.6 months in PRP group. The data in Table 1 shows the preoperative assessment of VAS, BCTQ-SSS, BCTQ-FSS, and grip strength, which showed no remarkable difference in demographics between the two groups.

**Table 1:** Patient features

	Control Group *N* = 39	PRP Group *N* = 43	*P*-value
**Gender**			
Male	17(43.6%)	18(41.8%)	0.874
Female	22 (56.4%)	25(58.2%)	
**Age**	42.5 ± 11.7	41.9 ± 11.7	0.839
**BMI**	25.6 ± 4.1	25.0 ± 5.1	0.608
**Affected side**			
Right	20 (51.2%)	26(60.4%)	0.402
Left	19(48.8%)	17(39.6%)	
**Symptom duration (month)**	24.3 ± 21.7	26.8 ± 23.6	0.613
**Grading**			
Moderate	26	26	0.560
Severe	13	17	
**VAS**	4.51 ± 1.27	4.58 ± 1.36	0.817
**BCTQ-SSS**	2.62 ± 0.51	2.80 ± 0.65	0.189
**BCTQ-FSS**	2.70 ± 0.68	2.86 ± 0.58	0.250
**Grip strength(g/mm**^**2**^**)**	16.74 ± 5.36	16.04 ± 5.15	0.556

*PRP* Platelet-rich plasma, *VAS* Visual analog scale, *BCTQ-SSS* Boston carpal tunnel syndrome questionnaire- symptom severity scale, *BCTQ-FSS* Boston carpal tunnel syndrome questionnaire- functional status scale

**Fig. 2:**
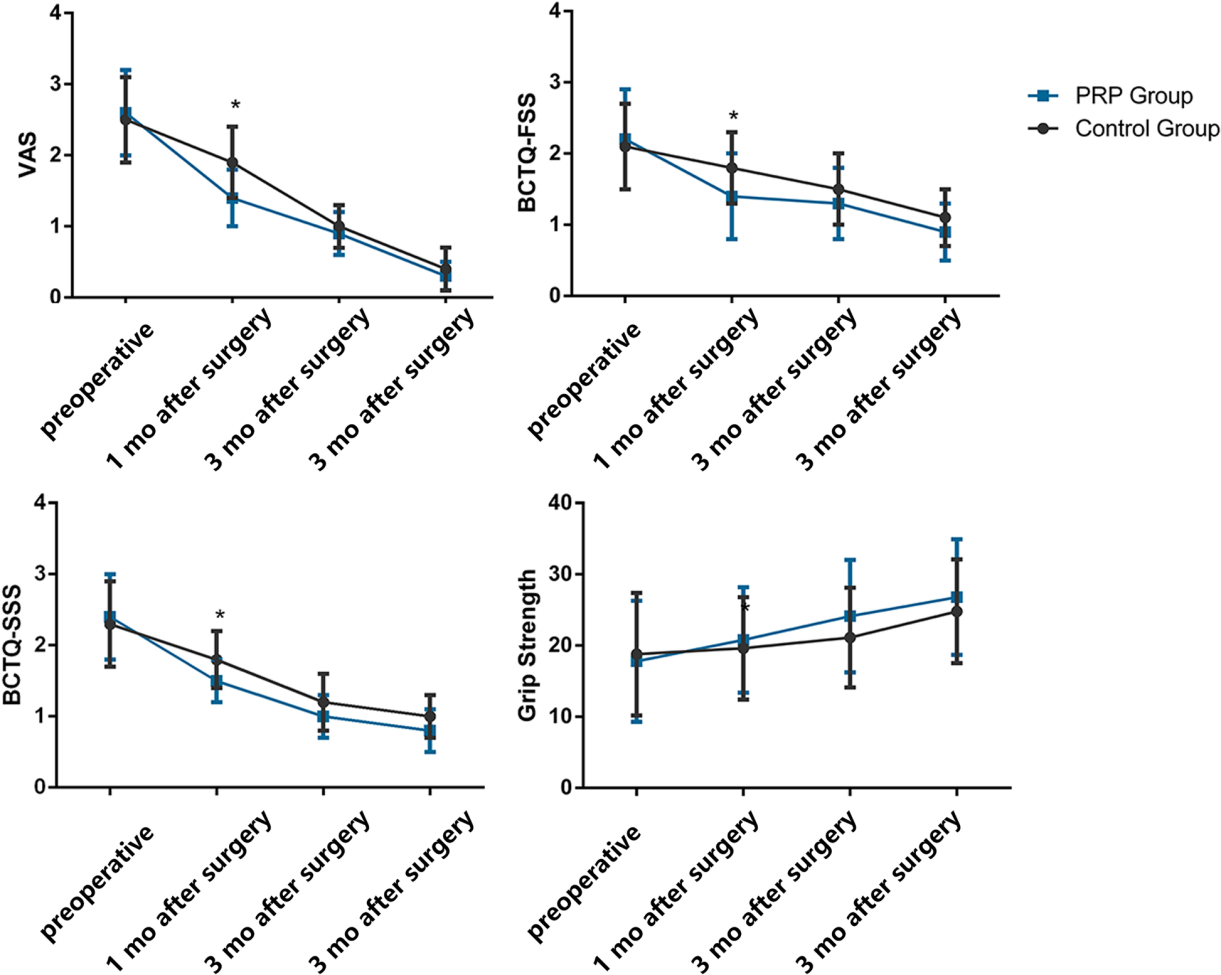
VAS BCTQ-FSS, BCTQ-SSS, and grips strength after surgery

The data in Table 2 shows the clinical outcomes within six months after surgery. The results showed no remarkable difference between the two groups in VAS, BCTQ-SSS, BCTQ-FSS, and grip strength, in 1 month, 3 months, and 6 months after surgery. However, the further analysis of patients with moderate preoperative symptoms showed that the PRP patient group showed a better outcome in VAS (2.42 ± 1.30 versus 1.69 ± 0.66, *P* < 0.05), BCTQ-SSS (1.90 ± 0.54 versus 1.57 ± 0.35, *P* < 0.05) and BCTQ-FSS (1.83 ± 0.57 versus 1.54 ± 0.37, *P* < 0.05) in 1 month after surgery. There was no marked difference between the two groups in VAS, BCTQ-SSS, BCTQ-FSS, and grip strength 3 months and 6 months after surgery (Table 3, Fig. 2).

**Table 2:** Clinical outcomes in all patients

	**1 month after surgery**			**3 months after surgery**			**6 months after surgery**		
	Control Group *N* = 39	PRP Group *N* = 43	P	Control Group *N* = 39	PRP Group *N* = 43	P	Control Group *N* = 39	PRP Group *N* = 43	P
**VAS**	2.61 ± 1.19	2.39 ± 1.18	0.410	1.38 ± 1.05	1.46 ± 1.16	0.747	0.71 ± 1.10	0.81 ± 1.16	0.707
**BCTQ-SSS**	2.13 ± 0.58	1.91 ± 0.59	0.097	1.46 ± 0.64	1.39 ± 0.67	0.630	1.02 ± 0.86	0.93 ± 0.85	0.665
**BCTQ-FSS**	2.11 ± 0.63	1.94 ± 0.78	0.290	1.60 ± 0.69	1.64 ± 0.71	0.824	1.14 ± 0.87	1.20 ± 0.83	0.740
**Grip strength (g/mm**^**2**^**)**	18.01 ± 5.24	17.88 ± 5.05	0.852	20.68 ± 5.15	21.81 ± 5.12	0.909	22.87 ± 5.10	24.34 ± 5.59	0.222

**Table 3:** Clinical outcomes in patients with moderate preoperative symptoms

	**Control Group** *N* = 26	**PRP Group** *N* = 26	P *Significant
**VAS** preoperative	4.00 ± 1.07	3.84 ± 1.16	0.630
**VAS** 1 month following surgery	2.42 ± 1.30	1.69 ± 0.66	0.016*
**VAS** 3 months following surgery	1.12 ± 0.93	1.00 ± 0.83	0.647
**VAS** 6 months following surgery	0.46 ± 0.69	0.42 ± 0.63	0.838
**BCTQ**-**SSS** preoperative	2.36 ± 0.41	2.41 ± 0.43	0.701
**BCTQ-SSS** 1 month following surgery	1.90 ± 0.54	1.57 ± 0.35	0.016*
**BCTQ-SSS** 3 months following surgery	1.25 ± 0.51	1.06 ± 0.48	0.201
**BCTQ-SSS** 6 months following surgery	0.80 ± 0.67	0.66 ± 0.61	0.438
**BCTQ-FSS** preoperative	2.42 ± 0.58	2.51 ± 0.41	0.521
**BCTQ-FSS** 1 month following surgery	1.83 ± 0.57	1.54 ± 0.37	0.037*
**BCTQ-FSS** 3 months following surgery	1.33 ± 0.56	1.29 ± 0.45	0.773
**BCTQ-FSS** 6 months following surgery	0.95 ± 0.73	0.87 ± 0.53	0.659
**Grip strength** preoperative (g/mm^2^)	19.47 ± 3.71	18.68 ± 4.36	0.470
**Grip strength** 1 month after surgery(g/mm^2^)	20.86 ± 3.53	20.44 ± 4.41	0.710
**Grip strength** 3 months after surgery (g/mm^2^)	23.19 ± 4.06	23.21 ± 4.87	0.983
**Grip strength** 6 months after surgery(g/mm^2^)	24.79 ± 4.04	26.25 ± 5.79	0.303

*Represents a statistical difference between two groups (*p* < 0.05)

The data in Table 4 showed that 35 (89.7%) and 40 (93%) individuals in the control and PRP groups revealed remission 6 months after surgery. 16 (41.0%) and 19(44.1%) individuals in the control and PRP groups were asymptomatic. Nonetheless, there was no remarkable difference between the two groups.

**Table 4:** Symptom Remission

	**Moderate Symptom**			**Severe Symptom**		
	**Control Group** *N* = 26	**PRP Group** *N* = 26	**P**	**Control Group** *N* = 13	**PRP Group** *N* = 17	**P**
**Remission**	25(96.1%)	26(100.0%)	0.312	10(76.9%)	14(82.4%)	0.712
**Non-remission**	1(3.9%)	0(0%)		3(23.1%)	3(17.6%)	
**Completely asymptomatic**	12(46.2%)	13(50.0%)	0.781	4(30.8%)	6(35.2%)	0.794

As shown in Table 5, the adjuvant PRP treatment increased the surgery costs (12,363.2 ± 901.2 versus 16,206.4 ± 1131.0, CNY, *P* < 0.001). There was no statistical difference in hospitalization between the two groups (3.56 ± 0.59 versus 3.46 ± 0.54 days, *P* = 0.29).

**Table 5:** Economic analysis

	**Control Group** *N* = 39	**PRP Group** *N* = 43	P *Significant
Hospitalization (Days)	3.56 ± 0.59	3.46 ± 0.54	0.437
Cost (CNY)	12,363.2 ± 901.2	16,206.4 ± 1131.0	< 0.001*

*CNY* China Yuan

*Represents a statistical difference between two groups (*p* < 0.05)

## Discussion

In this study, CTR combined with PRP treatment has been reported to improve the health outcomes and functions of patients with moderate symptoms within one month after surgery. Still, it does not show an advantage in treating individuals with severe carpal tunnel syndrome. At the same time, PRP's use does not affect long-term prognosis while increasing the surgery cost.

Some clinical and basic studies support PRP’s role in repairing peripheral nerve injuries, while it is beginning to be widely used in hand surgery [19, 20]. With the ongoing research on PRP treatment, different researchers have proposed that the effect of PRP in the CTS treatment is limited. Our study points out that in severe carpal tunnel syndrome, PRP combined with CTR does not improve the prognosis of patients, which may be related to the degeneration of the median nerve [21, 22]. In contrast, in mild to moderate carpal tunnel syndrome, adjuvant PRP therapy showed early therapeutic effects. A placebo-controlled clinical study indicated that a single PRP injection positively impacts individuals with mild to moderate CTS, supporting our findings [12]. In addition, some researchers believe that PRP injection did not add remarkably to the effects of a wrist splint. A randomized controlled trial pointed out that a single injection of PRP had no marked influence on the improvement effect of wrist splints in individuals with CTS [13]. However, a severe form of CTS was excluded in almost all PRP treatment studies for CTS, and treatment of PRP combined with CTR was not mentioned. In conclusion, many studies have indicated that PRP treatment is ineffective and unnecessary in patients with severe carpal tunnel syndrome and should be avoided in clinical practice [9].

For mild and moderate carpal tunnel syndrome, braking and control with oral medications are good options for treatment [23]. In contrast, for patients with severe carpal tunnel syndrome, the effectiveness of oral therapy alone is limited [5, 24]. CTR is still the most recommended treatment for individuals with severe carpal tunnel syndrome and some patients without improvement after glucocorticoid injection [5, 25, 26]. Adjuvant surgery has also achieved better treatment results [27–29]. This study concluded that CTR effectively treats persons with moderate or severe carpal tunnel syndrome, which can effectively improve the symptoms and functions, and even some patients' symptoms disappear entirely. The combined treatment of PRP does not increase the long-term effect of surgery, but it can accelerate the recovery after the operation in a short time.

Compared with many multicenter clinical studies, the number of subjects in this study is relatively small. Besides, the lack of randomization, blinding, and placebo constitutes another limitation of the present study, making the placebo effect unavoidable in this study. Issues apportioned into the PRP group but declined injection treatment were allowed to participate in the control group, which elevated the risk of selection bias. However, we believe that this bias is greatly reduced due to the surgery. In future studies, a large sample size of RCTs will help clarify the role of PRP in early clinical recovery and its effect on long-term treatment.

## Conclusion

To conclude, PRP as an adjuvant treatment of CTR has limited effect. Considering the additional financial burden on patients, CTR combined with PRP should be cautious in treating CTS.

## Data Availability

The final dataset will be available from the corresponding author.
